# Prognostic Index for Liver Radiation (PILiR)

**DOI:** 10.3390/curroncol31100436

**Published:** 2024-09-29

**Authors:** Laura Callan, Haddis Razeghi, Natalie Grindrod, Stewart Gaede, Eugene Wong, David Tan, Jason Vickress, John Patrick, Michael Lock

**Affiliations:** 1 BC Cancer-Kelowna, Kelowna, BC V1Y 5L3, Canada; laura.callan@bccancer.bc.ca; 2 Radiation Oncology, London Health Sciences Centre, London, ON N6A 5W9, Canada; srazeghi@uwo.ca (H.R.); ngrindro@uwo.ca (N.G.); stewart.gaede@lhsc.on.ca (S.G.); ewong4@uwo.ca (E.W.); jason.vickress@lhsc.on.ca (J.V.); johnc.patrick@lhsc.on.ca (J.P.); 3 Faculty of Nursing, Western University, London, ON N6A 3K7, Canada; 4 Pathology & Labaratory Medicine, Western University, London, ON N6A 3K7, Canada; 5 Department of Physics and Astronomy, Western University, London, ON N6A 3K7, Canada; 6 Asian Alliance Radiation & Oncology, Centre for Stereotactic Radiosurgery, Singapore 289891, Singapore; davidtan@aaro.sg; 7 Schulich School of Medicine, Western University, London, ON N6A 3K7, Canada

**Keywords:** hepatocellular carcinoma, nomogram, external beam radiotherapy, overall survival, stereotactic body radiotherapy, liver metastasis

## Abstract

A Prognostic Index for Liver Radiation (PILiR) for improved patient selection for stereotactic liver-directed radiotherapy (SBRT) was developed. Using a large single-center database, 195 patients treated with SBRT for local control, including 66 with hepatocellular carcinoma (HCC) and 129 with metastatic liver disease, were analyzed. Only patients ineligible for alternative treatments were included. Overall survival was 11.9 months and 9.4 months in the HCC group and metastatic groups, respectively. In the combined dataset, Child–Pugh Score (CPS) (*p* = 0.002), serum albumin (*p* = 0.039), and presence of extrahepatic disease (*p* = 0.012) were significant predictors of early death on multivariable analysis and were included in the PILiR (total score 0 to 5). Median survival was 23.8, 9.1, 4.5, and 2.6 months for patients with 0, 1–2, 3, and 4–5 points, respectively. In the HCC dataset, CPS (*p* < 0.001) and gross tumor volume (*p* = 0.013) were predictive of early death. In the metastatic dataset, serum albumin (*p* < 0.001) and primary disease site (*p* = 0.003) were predictive of early death. The AUC for the combined, HCC, and metastatic datasets are 0.78, 0.84, and 0.80, respectively. Poor liver function (defined by CPS and serum albumin) and extrahepatic disease were most predictive of early death, providing clinically important expected survival information for patients and caregivers.

## 1. Introduction

Malignant hepatic lesions are common and are associated with high mortality rates. The role of radiation therapy (RT) has historically been limited to palliation due to the risk of radiation-induced liver disease (RILD), which is of significant concern in a population that often has precarious hepatic function prior to treatment. With advances in delivery techniques and radiobiological understanding, dose-escalated RT can safely be delivered to more individuals, but the population that will benefit from this treatment has not yet been established.

The gold standard treatment of malignant liver lesions is surgery; however, less than 20% of patients are eligible for resection due to medical comorbidities, poor performance status, unresectable tumors, or patient refusal [1]. The less invasive local therapies of cryoablation, radiofrequency ablation (RFA), percutaneous ethanol injection, and transarterial chemoembolization (TACE) may offer a mild survival advantage, but are not always technically feasible [2,3]. RT is a non-invasive treatment option that has few clinical limitations and can be administered to most tumors and patients. Therefore, particularly as many referred patients have exhausted other options, there may be an inordinate pressure to treat it as the only option. However, many patients may not survive long enough to benefit from radiation due to early death or progression of disease outside of the liver. 

RT to the liver using modern techniques is generally well tolerated [4]. From the patient’s perspective, there can be a temporary worsening of appetite and fatigue, but quality of life measures generally return to baseline by 3 months [5]. Still, there remains a wide range in survival of patients in the literature, and it would be prudent to avoid irradiating patients who are unlikely to survive long enough to benefit from the treatment, thus saving them the potential short-term toxicities. The Child–Pugh Score [6], Barcelona Clinic Liver Cancer score (BCLC) [1], Model for End-Stage Liver Disease (MELD) [7], Albumin–Bilirubin model (ALBI) [8,9], and Cancer of the Liver Italian Program (CLIP) [7] are measures that have been developed to predict patient outcomes specific to liver disease; however, none of these were developed for patients being assessed for RT and none focused on identifying patients who should not receive radiation.

The aim of this study is to develop a Prognostic Index for Liver Radiation (PILiR) using easily and commonly obtained pre-treatment patient and tumor factors to improve patient selection for dose-escalated radiation treatment of liver malignancies. In particular, the focus of the model development is to identify patients for whom high-dose radiation should not be offered. This aim was achieved.

## 2. Materials and Methods

### 2.1. Patient Selection

A large, single institution hepatic lesion database from a tertiary cancer center with long term follow-up data was used to retrospectively collect data for analyses. IRBapproval was obtained for this study. Specifically, 212 patients who had undergone liver RT and were ineligible for alternative treatments were selected. Data were accessed for research purposes on the 3rd of January 2020. Inclusion criteria: Patients receiving liver-directed radiation between July 2004 and June 2014. Exclusion criteria: 800cGy or less in a single fraction. After applying these criteria, 195 patients were left for analysis.

### 2.2. Study Design and Statistical Analysis

Overall survival (OS) was calculated from the date of the first fraction of liver RT. Patients with an OS of 16 months or less were considered not to have benefitted from treatment. Patients with both curative intent and palliative intent were included in this study. This cutoff was chosen as a practical definition of early death after which patients were likely to have a better benefit-to-harm ratio, providing for a sufficient statistical balance.

The effect of baseline patient and tumor characteristics on OS was assessed using logistic regression. Univariable analysis was performed using commonly collected characteristics, listed in Table 1. Patient-related parameters included age, sex, CPS, serum albumin, total bilirubin, international normalized ratio (INR), and the clinical or radiographic presence of ascites. Tumor-related factors included gross tumor volume (GTV), number of hepatic lesions, presence of extrahepatic disease, previous therapy received (resection, chemotherapy, chemoembolization), and, in patients with metastatic liver lesions, primary disease site. Age, serum albumin, total bilirubin, INR, and GTV were treated as continuous variables. The number of hepatic lesions was treated as an ordinal variable. Sex, CPS (A vs. B/C), presence of ascites, previous therapy, and primary disease site (colorectal vs. other) were considered dichotomous variables.

Variables found to be significant (*p* < 0.05) on univariable analysis were then analyzed using a multivariable logistic regression. Validation of the multivariate model was done with a 3-fold cross-validation, performed with 500 counts of random splitting for bootstrapping. The results of validation were reported using the average area under the curve (AUC) from the receiver operator curve. A prognostic index was created using the odds ratios (ORs) of variables found to be significant on multivariable analysis.

This process was initially performed on the entire (combined) dataset and then repeated using the dataset composed of only those patients with hepatocellular carcinoma (HCC), and again using the dataset of patients with metastatic disease of the liver. R version 3.6.0. was used as the statistical software for this analysis.

## 3. Results

### 3.1. Patient and Tumor Characteristics

A total of 195 patients treated with liver-directed radiation between July 2004 and June 2014 were identified within a prospectively collected database from a single tertiary comprehensive cancer center. Patient characteristics at the initiation of treatment are indicated in Table 1. The median follow-up was 13.6 months (0.3–125.3). The mean age of the study population was 67 (22–89) years, with 123 (63%) males and 72 (37%) females. Of these, 66 patients (33.8%) had HCC and 129 (66.2%) had metastatic disease to the liver. Patients with cholangiocarcinoma were included within the metastatic group. For the metastatic group, primary disease sites included colorectal carcinoma (*n* = 52, 40.3%), cholangiocarcinoma (*n* = 22, 17.1%), neuroendocrine carcinoma (*n* = 10, 7.8%), breast carcinoma (*n* = 10, 7.8%), non-small cell lung carcinoma (*n* = 10, 7.8%), and others (*n* = 25, 19.4%) (Table 1). Radiation dose was chosen based on each patient’s Normal Tissue Complication Probability (NTCP) using the Lyman–Kutcher–Burman model for risk of RILD, similar to the method used for RTOG 1112 [10]. The mean dose was 4680 cGy (440–8775), and the average number of fractions was 10 (5–30). The mean equivalent dose in 2 Gy per fraction (EQD2) was 62 Gy (14.9–174.7), assuming an alpha/beta ratio of 10 Gy. The mean biologically effective dose (BED) was 84.1 Gy (17.9–209.7). Patients were treated with techniques of Volumetric Modulated Arc Therapy (VMAT), Intensity Modulated Radiation Therapy (IMRT), or Helical Tomotherapy (Tomo).

**Figure 1:**
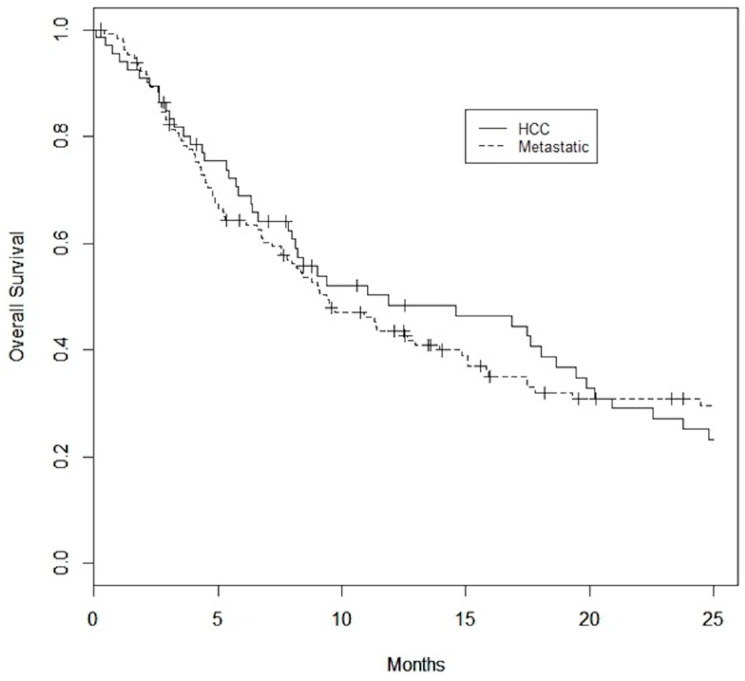
Kaplan–Meier Survival curves for HCC and metastatic datasets. Median overall survival is 11.9 months for the HCC dataset and 9.4 months for the metastatic dataset.

A total of 16 of 66 (24%) patients in the HCC group and 32 of 129 (25%) patients in the metastatic group were considered to have had early death. Median survival was 9.7 months (0.1–25.3), 11.9 (0.1–61.3) months, and 9.4 (0.3–125.3) months in the overall, HCC, and metastatic cohorts, respectively (Figure 1). Median follow-up was 13.6 months.

### 3.2. Prognostic Factors

#### 3.2.1. Combined Dataset

In the combined dataset, 154 patients died, and 41 patients were known to be alive at the end of the study period. On univariable analysis, baseline factors found to correlate with early death were CPS (OR 0.47, *p* < 0.001), serum albumin (OR 1.16, *p* < 0.001), total bilirubin (OR 0.97, *p* = 0.001), presence of ascites (OR 0.24, *p* = 0.001), GTV (OR 0.93, *p* = 0.011), presence of extrahepatic disease (OR 0.42, *p* = 0.015), previous liver resection (OR 6.41, *p* = 0.013), and primary colorectal carcinoma (OR 3.25, *p* = 0.021). On multivariable analysis, CPS, serum albumin, and presence of extrahepatic disease continued to be significant for predicting early death, with an AUC of 0.78 (Table 2).

#### 3.2.2. Hepatocellular Carcinoma Dataset

In the HCC dataset, 55 patients died, and 11 patients were known to be alive at the end of the study period. Baseline CPS (OR 0.44, *p* = 0.001), serum albumin (OR 1.15, *p* = 0.010), total bilirubin (OR 0.97, *p* = 0.039), presence of ascites (OR 0.25, *p* = 0.031), and GTV (OR 0.86, *p* = 0.030) had statistically significant impacts on the likelihood of early death on univariable analysis. CPS and GTV were found to be significant predictors of early death on multivariable analysis with an AUC of 0.84 (Table 2).

#### 3.2.3. Metastatic Dataset

In the metastatic dataset, 99 patients died, and 30 patients were known to be alive at the end of the study period. Baseline CPS (OR 0.46, *p* < 0.001), serum albumin (OR 1.19, *p* < 0.001), total bilirubin (OR 0.97, *p* = 0.016), presence of ascites (OR 0.17, *p* = 0.005), presence of extrahepatic disease (OR 0.34, *p* = 0.024), previous liver resection (OR 6.04, *p* = 0.019), and a primary colorectal malignancy (OR 3.98, *p* = 0.009) had statistically significant impacts on the likelihood of early death. Serum albumin and colorectal primary continued to be significant predictors on multivariable analysis with an AUC of 0.80 (Table 2).

### 3.3. PILiR Score

**Figure 2:**
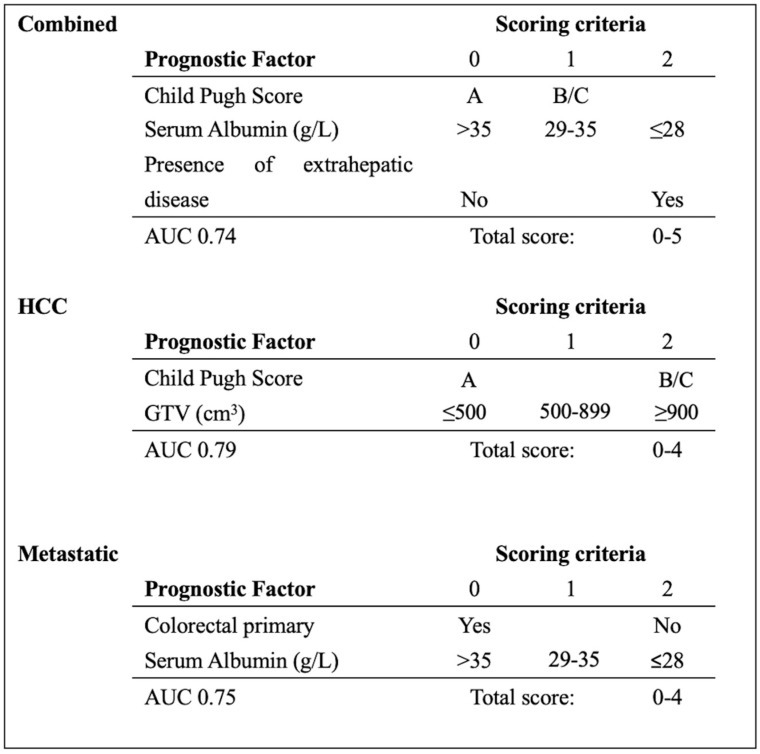
PILiR score to estimate survival based on pre-treatment characteristics. Median survival (months) in the combined group based on PILiR: 0 = 23.8; 1–2 = 9.1; 3 = 4.5; 4–5 = 2.6. Median survival (months) in the HCC group based on PILiR: 0 = 20.9; 1–3 = 7.8; 4 = 1.8. Median survival (months) in the metastatic group based on PILiR: 0 = 13.9; 1–2 = 13.0; 3–4 = 2.9.

**Figure 3:**
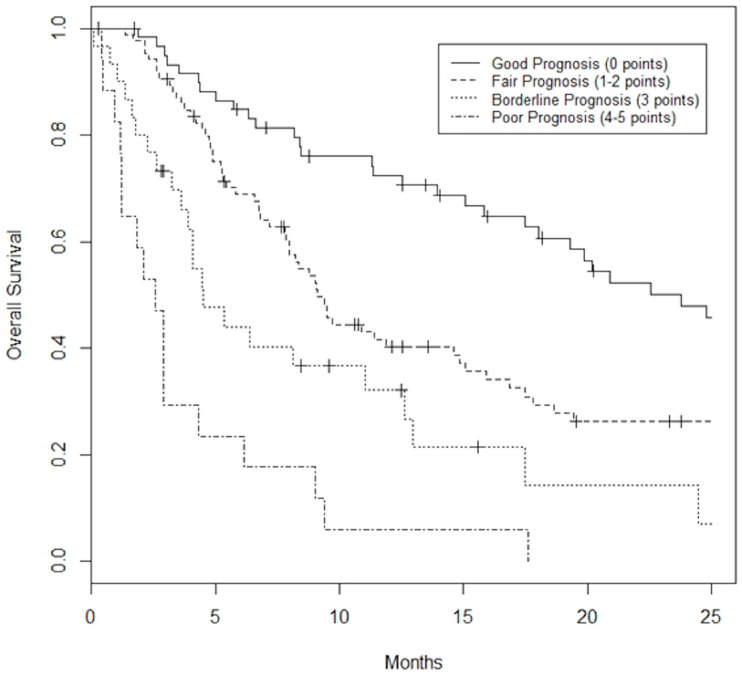
Kaplan–Meier Survival curves based on PILiR score for the combined dataset. Patients can be classified as Good Prognosis (0 points) with a median survival of 23.8 months, Fair Prognosis (1 or 2 points) with a median survival of 9.1 months, Borderline Prognosis (3 points) with a median survival of 4.5 months, or Poor Prognosis (4 or 5 points) with a median survival of 2.6 months.

**Figure 4:**
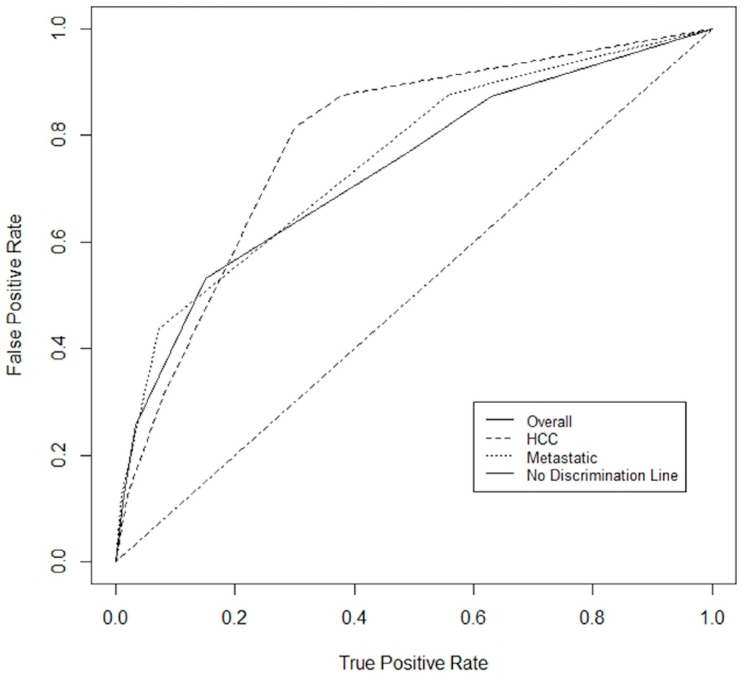
ROC for combined dataset, HCC dataset and metastatic dataset. The area under the curve (AUC) was 0.74, 0.79, and 0.75, respectively.

A prognostication tool was created for the combined dataset using weighting based on the odds radio from the multivariable analysis, rounding and applying clinically meaningful cutoffs to allow for ease of use. The PILiR score is defined in Figure 2. Patients were scored between 0 and 5. For our patient population, those with a score of 2 or less (*n* = 145, 75.6%) were found to have a median survival greater than 4 months and would therefore be likely to benefit from treatment. Those with a score of 4 or greater (*n* = 17, 8.8%) were found to have a median survival less than 4 months and would thereby be less likely to benefit from treatment. Patients with a score of 3 (*n* = 30, 15.5%) were found to have a survival of approximately 4 months and could therefore be considered borderline as to whether they would benefit from treatment by our definition (Table 3, Figure 3). The goal of the model was to provide strong discriminant ability to classify patients at risk for early death; therefore, cutoffs were chosen to maximize specificity to ensure patients who would benefit from treatment were not inappropriately included in the early death category. The specificity of this tool is 97% and the sensitivity is 26% using a decision-not-to-treat cutoff of 4 or greater points (Figure 4). Prognostic indices were also created for the HCC and metastatic datasets (Figure 2) with a resulting stratification of median overall survival, as described in Table 3. The AUC for the combined, HCC, and metastatic datasets are 0.74, 0.79, and 0.75, respectively. 

## 4. Discussion

Malignant liver lesions are common and a significant source of morbidity and mortality. While surgical resection is the gold standard, many patients are not candidates and alternative treatment options need to be considered. Radiotherapy is a valid alternative and is not limited by many factors that preclude other treatments such as percutaneous accessibility, adequate perfusion of the tumor, or procedural risks; however, it is not always clear who is most likely to benefit from this treatment since many of these patients have a guarded prognosis. Indeed, radiation may be the last option for many patients, and this may result in caregivers overtreating patients. An index using pre-treatment patient and tumor factors was created, allowing for improved patient selection by identifying patients who are more likely to experience early death and thereby would not benefit from dose-escalated radiotherapy.

In this study, the presence of extrahepatic disease and poor liver function, as indicated by a CPS of B or C and low serum albumin, were significant predictors of patients who did not benefit from treatment due to their poor prognosis, when considering all patients with malignant liver lesions (combined cohort). In patients with HCC, CPS and tumor volume were important predictive factors. In patients with metastatic liver disease, serum albumin and primary disease site were predictive. By using these factors, the PiLIR score was created (Figure 2), which has high specificity, allowing the clinician to ensure that patients with a short life expectancy do not receive unnecessary treatment. The tool is easy to use with routinely obtained clinical parameters that can provide immediate information at the time of consultation.

Validated tools for prognostication in patients with HCC exist, including the ALBI nomogram which uses liver function alone to predict survival [8], and the BCLC staging classification, which takes into account both patient factors (performance status and CPS) and disease factors (tumor size, number of tumors, vascular invasion, nodal spread, and presence of extrahepatic disease), and suggests that patients with early-stage disease benefit from curative intent with transplant, resection, or ablation; whereas those with intermediate or advanced disease benefit from chemoembolization or treatment on a clinical trial; and those with terminal disease benefit from supportive care [1]. Of these, only the ALBI has been validated for use in patients undergoing radiotherapy, but it does not specifically identify patients in which radiation should likely be avoided [8].

General nomograms have previously been created for patients undergoing liver SBRT. Kress et al. (2012) established a prognostic scoring system for use in patients with liver lesions undergoing SBRT where the presence of 2 or more lesions, active systemic disease, and poor performance status were found to be predictive of decreased overall survival [11]. This scoring system, however, is based on a small dataset of only 52 patients with heterogeneous disease histology. Furthermore, measures of hepatic function, which have previously been shown to correlate with patient outcomes [1,8], were not included in their analysis. Our nomogram established that factors such as CPS, serum albumin, total bilirubin, presence of ascites, GTV, presence of extrahepatic disease, previous liver resection, and primary colorectal carcinoma were predictive of OS.

Nomograms have also been created for patients with HCC undergoing radiation. Using the Surveillance, Epidemiology, and End Results Program (SEER) database, Zhan et al. (2023) created a model for patients with HCC treated with external beam radiotherapy (EBRT), where tumor size, TNM (tumor, nodal, metastasis) staging, AFP, previous surgery, and chemotherapy were predictive of OS [12]. Similar models have been developed [13,14,15]. These studies found multiple predictive factors including multiple lesions [14,15], tumor size [13,14,16,17,18,19,20,21,22,23], T (tumor) stage [14,17,18,21], N (nodal) stage [18], M (metastasis) stage [17,18,19,21,24], grade [17,18,23], tumor volume [16,23] American Joint Cancer Committee (AJCC) stages T3–4 [19], CPS [14,21], ECOG PS [20], ALBI grade [20], Portal Vein Tumor Thrombus (PVTT) classification [20], BCLC stage [20], macrovascular invasion [13,19], fibrosis score [17,19], histological type [18], presence of cirrhosis [14], biologic equivalent dose [13], AFP [14,15,16,18,19,20,23,25], C-reactive protein (CRP) [25,26], hemoglobin [14,25], platelets [15], alkaline phosphate [15,25], certain treatments [15,17,18,20,21,22,26], patient age [14,18,19], sex [18,21], race [18], year of diagnosis [18], symptoms related to lymph node metastasis [2], and uncontrolled intrahepatic disease [24].

The SEER database was also retrospectively used to create a nomogram for colorectal liver metastasis, using 9800 patients for training: 4897 patients for internal validation and 60 for external validation [27]. This nomogram had an AUC of 0.811 and 0.727 for the internal and external cohorts, respectively. Many factors were found to be predictive of OS in this nomogram and others, with liver metastases for pancreatic, esophageal, and non-small cell lung cancer, in addition to colorectal, acting as predictive factors [25,26,27,28,29,30,31,32]. These studies also found primary site [27,28], T stage [27,32], N stage [27,32], and metastasis of bone [27,28,30,32,33], brain [27,32,33], and lung [27,28,30,32,33] predictive of OS as well as multiple liver metastases [29], grade [28,30,32,33], AJCC N status [28], tumor size [28,30,32], histological type [28,30,33], chemotherapy [27,28,31,32,33], carcinoembryonic antigen (CEA) [28], poor differentiation [29,30], adenocarcinoma [30], and bilobar liver distribution [28]. Despite our current model incorporating a more heterogenous population than others mentioned above, the predictive AUC values are similar to theirs. The novelty of our nomogram is that it specifically aims to identify patients that would likely not benefit from radiation and for whom other management options should be sought.

There are important considerations and limitations to this study. First, in the current study, median overall survival was 11.9 months in the HCC group and 9.4 months in the metastatic group. This is shorter than other studies looking at survival in patients with malignant liver lesions, where median overall survival ranges from 14.5 months to 34 months [9,34,35,36,37,38]. However, patients included in our study were heavily pre-treated, with 126 out of 195 (64.6%) patients having undergone at least one previous treatment and 17 patients (8.7%) having undergone three or more previous treatment modalities. Therefore, the participants in this study likely exhibited a higher degree of treatment resistance and were in more advanced stages of disease. When compared against studies that included predominantly advanced HCC patients, such as in a trial of sorafenib in unresectable HCC where median overall survival was 9.3 months [8] and 1 year survival was 29% in untreated patients [1], our results were more in keeping with their survival data. Thus, clinicians who wish to use this nomogram should determine if the demographics of our patients are generalizable to theirs.

Second, this study was designed to create an index based on pre-treatment factors alone. We have published a separate analysis assessing the role of RT modality and dose distribution on prognosis, along with clinical nomograms for both HCC and liver metastasis patients for predicting overall survival [38].

Third, the current study produced a model based on analysis of one of the larger published databases of North American patients with malignant liver lesions treated with radiotherapy [39]. However, the dataset is quite heterogeneous with wide acceptance in parameters such as size of lesion, presence of thrombosis, and presence of extrahepatic disease. In this study, all metastatic patients with a primary disease site other than colorectal were grouped together, resulting in the pooling of diseases with varied prognoses. Other prognostic indices have stratified patients based on the primary disease site [40] which allows for more homogenous data and, therefore, better predictive ability; however, in doing so, the sample size decreases dramatically. A larger database will allow further stratification by primary disease site, allowing for improved predictive ability.

The PILiR scoring system is designed to identify patients that are unlikely to benefit from radiation, and the goal was to have a high specificity to avoid incorrectly categorizing patients who would then likely receive only supportive care and be inappropriately excluded from the opportunity to receive a beneficial treatment. However, to avoid this type of error, this nomogram has low sensitivity. Compared to the total population, the number of patients living less than 4 months was relatively low, which resulted in a small number of patients in the higher point groups. This was especially true for the subgroup indices where there were only 3 patients in the HCC subgroup scoring 4 points, and only 5 patients in the metastatic subgroup scoring 4 points. By analyzing a larger, more homogenous population, an index with improved specificity and sensitivity can be created. A larger population will also allow us to provide more focused information, such as information assessing the value of treatment for oligometastases, oligoprogression, and dominant lesion control. Currently, these patients are grouped into one category with varying goals of treatment. 

Use of the PiLiR scoring system can allow for identification of patients who could be considered for aggressive radiotherapy for longer term local control versus patients who may instead benefit from a focus on short-term symptom control alone. By screening at consultation with this tool, patients who would best be served by palliation due to poor life expectancy, as indicated by a high PILiR score, can be identified. In such cases, radiotherapy with palliative dosing, such as 800cGy delivered in a single fraction, has been shown to improve patient-reported symptoms and quality of life at 1 month post-treatment [41,42]. On the other hand, those with low PILiR scores should be considered for aggressive radiation treatment with the goal of controlling tumor progression and future burden of disease.

A large, shared database across several cancer centers is being developed which will allow for further analysis and validation with higher power and reduced bias. External validation will allow for further confidence that the scoring system can provide clinicians with the tools to select patients appropriately and reliably for radiation. A future study could also include a randomized control trial or matched cohort design to determine if the radiation had an effect on survival.

## 5. Conclusions

A clinical prognostication tool has been created which allows for better identification of patients who likely should not receive dose-escalated RT. Following validation with a larger dataset and external validation, this tool will allow for better selection of patients for liver radiation.

## Figures and Tables

**Table 1:** Patient characteristics.

Characteristic	HCC	Mets
	(*N* = 66)	(*N* = 129)
**Age (years)**	70 ± 12	65 ± 12
**Sex**		
Male	56 (85%)	67 (52%)
Female	10 (15%)	62 (48%)
**Gross Tumor Volume (cm^3^)**	384 ± 447	351 ± 870
**Number of Lesions**		
1	45 (68%)	65 (50%)
2	7 (11%)	32 (25%)
3	10 (15%)	19 (15%)
>3	4 (6%)	13 (10%)
**Child–Pugh Score**		
A	42 (64%)	99 (77%)
B	22 (33%)	28 (22%)
C	2 (3%)	2 (1%)
**Bilirubin (μmol/L)**	21.6 ± 29	17 ± 32.7
**Serum Albumin (g/L)**	35 ± 6	38 ± 6
**INR**	1.36 ± 0.79	1.22 ± 0.47
**Extrahepatic Disease**		
Y	16 (24.2%)	73 (56.6%)
N	50 (75.8%)	56 (43.4%)
**Ascites**		
Y	19 (29%)	12 (9%)
N	47 (71%)	117 (91%)
**Primary Site (*N* = 129)**		
colorectal carcinoma		52 (40%)
Cholangiocarcinoma		22 (17%)
neuroendocrine carcinoma		10 (8%)
breast carcinoma		10 (8%)
non-small cell lung carcinoma		10 (8%)
pancreatic carcinoma		7 (5%)
renal cell carcinoma		3 (2%)
metastatic melanoma		3 (2%)
gastric carcinoma		3 (2%)
esophageal		3 (2%)
Other		6 (4%)
**Previous Treatments**		
chemo embolization	17 (26%)	7 (5%)
I-131 lipiodol	7 (11%)	6 (5%)
radiofrequency ablation	3 (5%)	9 (7%)
chemotherapy	6 (9%)	95 (74%)
liver resection	5 (8%)	32 (25%)

**Table 2:** Multivariable analysis.

**Combined**	**Parameter**	**OR**	**95% CI**	***p* Value**
	Child–Pugh Score	0.6	[0.40–0.82]	0.002
	Serum Albumin (g/L)	1.1	[1.00–1.18]	0.039
	Extrahepatic disease	0.4	[0.16–0.80]	0.012
	AUC 0.78			
**HCC**	**Parameter**	**OR**	**95% CI**	***p* Value**
	Child–Pugh Score	0.4	[0.22–0.66]	<0.001
	GTV (100 cm^3^)	0.8	[0.70–0.96]	0.013
	AUC 0.84			
**Metastatic**	**Parameter**	**OR**	**95% CI**	***p* Value**
	Serum Albumin (g/L)	1.2	[1.11–1.36]	<0.001
	Colorectal primary	6.7	[1.90–23.46]	0.003
	AUC 0.80			

**Table 3:** Survival based on PILiR score.

**Combined**	**Score**	***N***	**Median Survival (Months)**	**Percent Living Past 4 Months**
	0	60	23.8	90.0
	1	26	9.0	80.1
	2	60	9.4	81.7
	3	30	4.5	56.7
	4	9	1.8	33.3
	5	8	2.7	25.0
**HCC**	**Score**	***N***	**Median Survival (Months)**	**Percent Living Past 4 Months**
	0	33	20.9	93.9
	1	5	7.8	80.0
	2	19	5.7	57.9
	3	6	8.1	50.0
	4	3	1.8	33.3
**Metastatic**	**Score**	***N***	**Median Survival (Months)**	**Percent Living Past 4 Months**
	0	36	13.9	91.7
	1	11	9.4	90.9
	2	61	14.9	77.0
	3	16	3.7	37.5
	4	5	2.1	20.0

PILiR score to estimate survival based on pre-treatment characteristics. Median survival (months) in the combined group based on PILiR score: 0 = 23.8; 1–2 = 9.1; 3 = 4.5; 4–5 = 2.6. Median survival (months) in the HCC group based on PILiR score: 0 = 20.9; 1–3 = 7.8; 4 = 1.8. Median survival (months) in the metastatic group based on PILiR Score: 0 = 13.9; 1–2 = 13.0; 3–4 = 2.9.

## Data Availability

Data can be made available upon request.

## References

[1] Llovet J.M., Fuster J., Bruix J., Barcelona-Clínic Liver Cancer Group (2004). The Barcelona approach: Diagnosis, staging, and treatment of hepatocellular carcinoma. Liver Transpl..

[2] Lo C.M., Ngan H., Tso W.K., Liu C.L., Lam C.M., Poon T.P., Fan S.T., Wong J. (2002). Randomized controlled trial of transarterial lipiodol chemoembolization for unresectable hepatocellular carcinoma. Hepatology.

[3] Llovet J.M., Real M.I., Montaña X., Planas R., Coll S., Aponte J., Ayuso C., Sala M., Muchart J., Sola R. (2002). Arterial embolisation or chemoembolisation versus symptomatic treatment in patients with unresectable hepatocellular carcinoma: A randomised controlled trial. Lancet.

[4] Wahl D.R., Stenmark M.H., Tao Y., Pollom E.L., Caoili E.M., Lawrence T.S., Schipper M.J., Feng M. (2016). Outcomes After Stereotactic Body Radiotherapy or Radiofrequency Ablation for Hepatocellular Carcinoma. J. Clin. Oncol..

[5] Klein J., Dawson L.A., Jiang H., Kim J., Dinniwell R., Brierley J., Wong R., Lockwood G., Ringash J. (2015). Prospective Longitudinal Assessment of Quality of Life for Liver Cancer Patients Treated With Stereotactic Body Radiation Therapy. Int. J. Radiat. Oncol. Biol. Phys..

[6] Pugh R.N., Murray-Lyon I.M., Dawson J.L., Pietroni M.C., Williams R. (1973). Transection of the oesophagus for bleeding oesophageal varices. Br. J. Surg..

[7] Malinchoc M., Kamath P.S., Gordon F.D., Peine C.J., Rank J., ter Borg P.J., Pieter C.J. (2000). A model to predict poor survival in patients undergoing transjugular intrahepatic portosystemic shunts. Hepatology.

[8] Johnson P.J., Berhane S., Kagebayashi C., Satomura S., Teng M., Reeves H.L., O’Beirne J., Fox R., Skowronska A., Palmer D. (2015). Assessment of liver function in patients with hepatocellular carcinoma: A new evidence-based approach-the ALBI grade. J. Clin. Oncol..

[9] Lo C.H., Liu M.Y., Lee M.S., Yang J.F., Jen Y.M., Lin C.S., Chao H.L., Shen P.C., Huang W.Y. (2017). Comparison Between Child-Turcotte-Pugh and Albumin-Bilirubin Scores in Assessing the Prognosis of Hepatocellular Carcinoma After Stereotactic Ablative Radiation Therapy. Int. J. Radiat. Oncol. Biol. Phys..

[10] Dawson L.A., Normolle D., Balter J.M., McGinn C.J., Lawrence T.S., Tenhaken R.K. (2002). Analysis of radiation-induced liver disease using the Lyman NTCP model. Int. J. Radiat. Oncol. Biol. Phys..

[11] Kress M.A., Collins B.T., Collins S.P., Dritschilo A., Gagnon G., Unger K. (2012). Scoring system predictive of survival for patients undergoing stereotactic body radiation therapy for liver tumors. Radiat. Oncol..

[12] Zhan G., Peng H., Zhou L., Jin L., Xie X., He Y., Wang X., Du Z., Cao P. (2023). A web-based nomogram model for predicting the overall survival of hepatocellular carcinoma patients with external beam radiation therapy: A population study based on SEER database and a Chinese cohort. Front. Endocrinol..

[13] Huang W.Y., Tsai C.L., Que J.Y., Lo C.H., Lin Y.J., Dai Y.H., Yang J.F., Shen P.C., Lee M.H., Cheng J.H. (2020). Development and Validation of a Nomogram for Patients with Nonmetastatic BCLC Stage C Hepatocellular Carcinoma after Stereotactic Body Radiotherapy. Liver Cancer.

[14] Li X., Ye Z., Lin S., Pang H. (2021). Predictive factors for survival following stereotactic body radiotherapy for hepatocellular carcinoma with portal vein tumour thrombosis and construction of a nomogram. BMC Cancer.

[15] Long M., Li J., He M., Qiu J., Zhang R., Liu Y., Liang C., Lu H., Pang Y., Zhou H. (2023). Establishment and validation of a prognostic pomogram in unresectable hepatocellular carcinoma treated with intensity modulated radiotherapy: A real world study. Radiat. Oncol..

[16] Dewas S., Bibault J.E., Mirabel X., Fumagalli I., Kramar A., Jarraya H., Larcornerie T., Dewas-Vautravers C., Lartigau E. (2012). Prognostic factors affecting local control of hepatic tumors treated by Stereotactic Body Radiation Therapy. Radiat. Oncol..

[17] Zhang K., Tao C., Wu F., Wu J., Rong W. (2021). A practical nomogram from the SEER database to predict the prognosis of hepatocellular carcinoma in patients with lymph node metastasis. Ann. Palliat. Med..

[18] Li F., Zheng T., Gu X. (2022). Prognostic risk factor analysis and nomogram construction for primary liver cancer in elderly patients based on SEER database. BMJ Open.

[19] Zhang R., Chen J., Jiang Y., Wang J., Chen S. (2020). Prognostic nomogram for hepatocellular carcinoma with fibrosis of varying degrees: A retrospective cohort study. Ann. Transl. Med..

[20] Kim N., Yu J.I., Park H.C., Hong J.Y., Lim H.Y., Goh M.J., Paik Y.H. (2023). Nomogram for predicting overall survival in patients with large (>5 cm) hepatocellular carcinoma based on real-world practice. J. Liver Cancer.

[21] Zhou Y., Zhou X., Ma J., Zhang W., Yan Z., Luo J. (2021). Nomogram for Predicting the Prognosis of Patients with Hepatocellular Carcinoma Presenting with Pulmonary Metastasis. Cancer Manag. Res..

[22] Fendler W.P., Ilhan H., Paprottka P.M., Jakobs T.F., Heinemann V., Bartenstein P., Khalaf F., Ezziddin S., Hacker M., Haug A.R. (2015). Nomogram including pretherapeutic parameters for prediction of survival after SIRT of hepatic metastases from colorectal cancer. Eur. Radiol..

[23] Pan Q.Z., Wang Q.J., Dan J.Q., Pan K., Li Y.Q., Zhang Y.J., Zhao J.J., Weng D.S., Tang Y., Huang L.X. (2015). A nomogram for predicting the benefit of adjuvant cytokine-induced killer cell immunotherapy in patients with hepatocellular carcinoma. Sci. Rep..

[24] Wee C.W., Kim K., Chie E.K., Yu S.J., Kim Y.J., Yoon J.H. (2016). Prognostic stratification and nomogram for survival prediction in hepatocellular carcinoma patients treated with radiotherapy for lymph node metastasis. Br. J. Radiol..

[25] Chicco D., Oneto L. (2021). Computational intelligence identifies alkaline phosphatase (ALP), alpha-fetoprotein (AFP), and hemoglobin levels as most predictive survival factors for hepatocellular carcinoma. Health Inform. J..

[26] Scheiner B., Pomej K., Kirstein M.M., Hucke F., Finkelmeier F., Waidmann O., Himmelsbach V., Schulze K., von Felden J., Frundt T.W. (2022). Prognosis of patients with hepatocellular carcinoma treated with immunotherapy—Development and validation of the CRAFITY score. J. Hepatol..

[27] Kuai L., Zhang Y., Luo Y., Li W., Li X.D., Zhang H.P., Liu T.Y., Yin S.Y., Li B. (2021). Prognostic Nomogram for Liver Metastatic Colon Cancer Based on Histological Type, Tumor Differentiation, and Tumor Deposit: A TRIPOD Compliant Large-Scale Survival Study. Front. Oncol..

[28] Liu C., Hu C., Huang J., Xiang K., Li Z., Qu J., Liu Y., Zhang G., Wen T. (2021). A Prognostic Nomogram of Colon Cancer With Liver Metastasis: A Study of the US SEER Database and a Chinese Cohort. Front. Oncol..

[29] Yao J., Chen Q., Deng Y., Zhao J., Bi X., Li Z., Huang Z., Zhang Y., Zhou J., Zhao H. (2021). Nomograms predicting primary lymph node metastases and prognosis for synchronous colorectal liver metastasis with simultaneous resection of colorectal cancer and liver metastases. Ann. Palliat. Med..

[30] Shi H., Li X., Chen Z., Jiang W., Dong S., He R., Zhou W. (2023). Nomograms for Predicting the Risk and Prognosis of Liver Metastases in Pancreatic Cancer: A Population-Based Analysis. J. Pers. Med..

[31] Cao B.Y., Tong F., Zhang L.T., Kang Y.X., Wu C.C., Wang Q.Q., Yang W., Wang J. (2023). Risk factors, prognostic predictors, and nomograms for pancreatic cancer patients with initially diagnosed synchronous liver metastasis. World J. Gastrointest. Oncol..

[32] Zhao R., Dai Y., Li X., Zhu C. (2022). Construction and validation of a nomogram for non small cell lung cancer patients with liver metastases based on a population analysis. Sci. Rep..

[33] Xiong H., Hu D., Li M., Chen T., Jiang W., Xiang Z. (2022). Epidemiology and prognostic nomogram for esophageal cancer with liver metastasis based on the Surveillance, Epidemiology, and End Results database. Res. Sq..

[34] Van der Pool A.E., Méndez-Romero A., Wunderink W., Heijmen B.J., Levendag P.C., Verhoef C., Ijzermans J.N. (2010). Stereotactic body radiation therapy for colorectal liver metastases. Br. J. Surg..

[35] Katz A.W., Carey-Sampson M., Muhs A.G., Milano M.T., Schell M.C., Okunieff P. (2007). Hypofractionated stereotactic body radiation therapy (SBRT) for limited hepatic metastases. Int. J. Radiat. Oncol. Biol. Phys..

[36] Rusthoven K.E., Kavanagh B.D., Gaspar L.E., Schefter T.E., Cardenes H., Stieber V.W., Burn S.H., Feigenberg S.J., Chidel M.A., Pugh T.J. (2009). Multi-institutional phase I/II trial of stereotactic body radiation therapy for liver metastases. J. Clin. Oncol..

[37] Lee M.T., Kim J.J., Dawson L.A., Dinniwell R., Brierley J., Lockwood G., Wong R., Cummings B., Ringash J., Tse R.V. (2009). Phase I study of individualized stereotactic body radiotherapy of liver metastases. J. Clin. Oncol..

[38] Vickress J., Lock M., Lo S., Gaede S., Leung A., Cao J., Barnett R., Yartsev S. (2017). A multivariable model to predict survival for patients with hepatic carcinoma or liver metastasis receiving radiotherapy. Future Oncol..

[39] Kalogeridi M.A., Zygogianni A., Kyrgias G., Kouvaris J., Chatziioannou S., Kelekis N., Kouloulias V. (2015). Role of radiotherapy in the management of hepatocellular carcinoma: A systematic review. World J. Hepatol..

[40] Sperduto P.W., Kased N., Bhatt A., Jensen A.W., Brown P.D., Shih H., Kirkpatrick J., Gaspar L.E., Fiveash J.B., Chiang V. (2012). Summary report on the graded prognostic assessment: An accurate and facile diagnosis-specific tool to estimate survival for patients with brain metastases. J. Clin. Oncol..

[41] Soliman H., Ringash J., Jiang H., Singh K., Kim J., Dinniwell R., Brade A., Wong R., Brierly J., Cummings B. (2013). Phase II trial of palliative radiotherapy for hepatocellular carcinoma and liver metastases. J. Clin. Oncol..

[42] Dawson L.A., Fairchild A.M., Dennis K., Mahmud A., Stuckless T.L., Vincent F., Roberge D., Follwell M., Wong R., Jonker D.J. (2023). Canadian Cancer Trials Group HE.1: A phase III study of palliative radiotherapy for symptomatic hepatocellular carcinoma and liver metastases. J. Clin. Oncol..

